# Supplementary material for: A UMLS-based spell checker for natural language processing in vaccine safety
Source: BMC Med Inform Decis Mak. 2007 Feb 12;7:3. doi: 10.1186/1472-6947-7-3 (PMC1805499; doi:10.1186/1472-6947-7-3)
Supplement: Additional file 13 — RAP application directory. Sets up directory for RAP (RDF application for PHP) source code [file 1472-6947-7-3-S13.gz › rap/doc/phpdoc/elementindex_infModel.html]

xml version="1.0" encoding="iso-8859-1"?


## [infModel] element index

### Package indexes

- model
- dataset
- resModel
- ontModel
- syntax
- rdql
- utility

All elements
  

a
c
e
f
g
i
l
r
s
t
w

a

top

add
:   InfModel::add() in InfModel.php

    Adds a new triple to the Model without checking if the statement is already in the Model.

add
:   InfModelF::add() in InfModelF.php

    Adds a new triple to the MemModel without checking if the statement is already in the MemModel.

add
:   InfModelB::add() in InfModelB.php

    Adds a new triple to the Model without checking, if the statement is already in the Model. So if you want a duplicate free Model use the addWithoutDuplicates() function (which is slower then add())

addModel
:   InfModelF::addModel() in InfModelF.php

    Adds another model to this MemModel.

addWithoutDuplicates
:   InfModelF::addWithoutDuplicates() in InfModelF.php

    Checks if a new statement is already in the MemModel and adds the statement, if it is not in the MemModel.

c

top

checkEntailment
:   InfRule::checkEntailment() in InfRule.php

    Checks, if this rule could entail a statement that matches

checkTrigger
:   InfRule::checkTrigger() in InfRule.php

    Checks, if the statement satisfies the trigger

contains
:   InfModelB::contains() in InfModelB.php

    Tests if the Model contains the given triple.

e

top

entail
:   InfRule::entail() in InfRule.php

    Returns a infered InfStatement by evaluating the statement with the entailment rule.

entailStatement
:   InfModelF::entailStatement() in InfModelF.php

    Entails a statement by recursively using the \_entailStatementRec method.

f

top

find
:   InfModelB::find() in InfModelB.php

    General method to search for triples.

findAsIterator
:   InfModelB::findAsIterator() in InfModelB.php

    Returns a FindIterator for traversing the MemModel.

findFirstMatchingStatement
:   InfModelB::findFirstMatchingStatement() in InfModelB.php

    Searches for triples and returns the first matching statement.

findRegex
:   InfModel::findRegex() in InfModel.php

    Method to search for triples using Perl-style regular expressions.

findVocabulary
:   InfModel::findVocabulary() in InfModel.php

    Returns all tripels of a certain vocabulary.

g

top

getBaseMemModel
:   InfModelB::getBaseMemModel() in InfModelB.php

    Create a MemModel containing only the base triples (without inferred statements) of the current InfModelB

getBaseMemModel
:   InfModelF::getBaseMemModel() in InfModelF.php

    Create a MemModel containing only the base triples (without inferred statements) of the current InfModelF

getEntailment
:   InfRule::getEntailment() in InfRule.php

getMemModel
:   InfModelB::getMemModel() in InfModelB.php

    Create a MemModel containing all the triples (including inferred statements) of the current InfModelB

getMemModel
:   InfModelF::getMemModel() in InfModelF.php

    Create a MemModel containing all the triples (including inferred statements) of the current InfModelF

getModifiedFind
:   InfRule::getModifiedFind() in InfRule.php

    Returns a find-query that matches statements, whose entailed statements would match the supplied find query.

getSchema
:   InfModel::getSchema() in InfModel.php

    Returns a model, containing all Statements, having a Predicate, that is supported by the inference.

getStatementIterator
:   InfModelB::getStatementIterator() in InfModelB.php

    Returns a StatementIterator for traversing the Model.

getTrigger
:   InfRule::getTrigger() in InfRule.php

i

top

InfModel
:   InfModel in InfModel.php

    A InfModel Model extends a MemModel , by adding the ability to infer statements from known statements and RDFS/OWL-Schematas.

InfModel
:   InfModel::InfModel() in InfModel.php

    Constructor You can supply a base\_uri

InfModelB
:   InfModelB::InfModelB() in InfModelB.php

    Constructor You can supply a base\_uri

InfModelB
:   InfModelB in InfModelB.php

    A InfModelB extends the InfModel Class, with a backward chaining algorithm.

InfModelF
:   InfModelF in InfModelF.php

    A InfModelF extends the InfModel Class, with a forward chaining algorithm.

InfModelF
:   InfModelF::InfModelF() in InfModelF.php

    Constructor You can supply a base\_uri

InfModelB.php
:   InfModelB.php in InfModelB.php

InfModelF.php
:   InfModelF.php in InfModelF.php

InfModel.php
:   InfModel.php in InfModel.php

InfRule.php
:   InfRule.php in InfRule.php

InfStatement.php
:   InfStatement.php in InfStatement.php

InfRule
:   InfRule in InfRule.php

    This class represents a single rule in a RDFS inference model.

infRule
:   InfRule::infRule() in InfRule.php

    Constructor

InfStatement
:   InfStatement in InfStatement.php

    An RDF statement which was entailed by a inference rule.

l

top

load
:   InfModelF::load() in InfModelF.php

    Load a model from a file containing RDF, N3 or N-Triples.

r

top

remove
:   InfModelF::remove() in InfModelF.php

    Removes the triple from the MemModel.

remove
:   InfModelB::remove() in InfModelB.php

    Removes the triple from the MemModel.

removeInfered
:   InfModelF::removeInfered() in InfModelF.php

    Removes all infered statements from the model but keeps the infernece rules.

replace
:   InfModel::replace() in InfModel.php

    General method to replace nodes of a MemModel.

s

top

saveAs
:   InfModelB::saveAs() in InfModelB.php

    Saves the RDF,N3 or N-Triple serialization of the full InfModelB (including inferred triples) to a file.

setEntailment
:   InfRule::setEntailment() in InfRule.php

    Sets the entailment of this rule The values can be NULL to match anything or be a node that has to be matched.

setTrigger
:   InfRule::setTrigger() in InfRule.php

    Sets the trigger of this rule The values can be NULL to match anything or be a node that has to be matched.

size
:   InfModelB::size() in InfModelB.php

    Number of all inferable triples in the Model.

t

top

toString
:   InfModelB::toString() in InfModelB.php

    Short Dump of the InfModelB.

toString
:   InfModelF::toString() in InfModelF.php

    Short Dump of the InfModelF.

toStringIncludingTriples
:   InfModelB::toStringIncludingTriples() in InfModelB.php

    Dumps of the InfModelB including ALL inferable triples.

w

top

writeAsHtml
:   InfModelB::writeAsHtml() in InfModelB.php

    Writes the RDF serialization of the Model including ALL inferable triples as HTML.

writeAsHtmlTable
:   InfModelB::writeAsHtmlTable() in InfModelB.php

    Writes the RDF serialization of the Model including ALL inferable triples as HTML table.

writeRdfToString
:   InfModelB::writeRdfToString() in InfModelB.php

    Writes the RDF serialization of the Model including ALL inferable triples.

a
c
e
f
g
i
l
r
s
t
w
